# Early Virus-Host Interactions Dictate the Course of a Persistent Infection

**DOI:** 10.1371/journal.ppat.1004588

**Published:** 2015-01-08

**Authors:** Brian M. Sullivan, John R. Teijaro, Juan Carlos de la Torre, Michael B. A. Oldstone

**Affiliations:** 1 Viral-Immunobiology Laboratory, Department of Immunology and Microbial Science, The Scripps Research Institute, La Jolla, California, United States of America; University of Southern California, United States of America

## Abstract

Many persistent viral infections are characterized by a hypofunctional T cell response and the upregulation of negative immune regulators. These events occur days after the initiation of infection. However, the very early host-virus interactions that determine the establishment of viral persistence remain poorly uncharacterized. Here we show that to establish persistence, LCMV must counteract an innate anti-viral immune response within eight hours after infection. While the virus triggers cytoplasmic RNA sensing pathways soon after infection, LCMV counteracts this pathway through a rapid increase in viral titers leading to a dysfunctional immune response characterized by a high cytokine and chemokine expression profile. This altered immune environment allows for viral replication in the splenic white pulp as well as infection of immune cells essential to an effective anti-viral immune response. Our findings illustrate how early events during infection critically dictate the characteristics of the immune response to infection and facilitate either virus control and clearance or persistence.

## Author Summary

Lymphocytic Choriomenengitis Virus (LCMV) is an important model for the investigation of the pathogenesis of persistent viral infections. As with humans infected with hepatitis C and Human Immunodeficiency Virus-1, adult mice persistently infected with immunosuppressive strains of LCMV express high levels of negative immune regulators that suppress the adaptive T cell immune response thereby facilitating viral persistence. Unknown, however, is whether and how very early interactions between the virus and the infected host affect the establishment of a persistent infection. Here, we describe host-virus interactions within the first 8–12 hours of infection are critical for establishing a persistent infection. While early induction of an anti-viral type-I interferons is essential for the subsequent adaptive immune response required to clear the virus, LCMV is able to overcome the programmed innate immune response by over-stimulating this response early. This affects not only the rate of viral growth in the host, but also the ability to infect specific immune cells that help shape an effective adaptive immune response. We further describe how and where LCMV is sensed by this early immune response, identify the critical timing of early virus-host interactions that lead to a persistent infection, and identify an early dysregulated immune signature associated with a persistent viral infection. Altogether, these observations are critical to understanding how early virus-host interactions determines the course of infection.

## Introduction

The innate antiviral immune response is primarily triggered by recognition of virally derived molecules, a.k.a. pathogen associated molecular patterns (PAMPs), by host cell pathogen recognition receptors (PRR), resulting in the induction of type-I interferons (IFN-I), a group of molecules that exhibit potent anti-viral properties and also contribute to the expansion and survival of specific anti-viral cytotoxic T lymphocytes [1]–[4]. Accordingly, viruses have evolved a plethora of mechanisms to counteract the induction of IFN-I and downstream events triggered by IFN-I signaling [5]–[9], which often play critical roles in virulence [8], [10]–[13]. Similar to many other viruses, although LCMV infection induces a strong IFN-I response, it also encodes proteins that counteract the induction of IFN-I [14]–[17]. Notably, we [18] and others [19] have recently reported that, unexpectedly, IFN-I induced early during infection of mice with the immunosuppressive strain clone 13 (Cl13) of LCMV plays a critical role in the establishment of Cl13 persistence. These findings illustrate how IFN-I can both hamper and promote virus infection. Thus, in the case of LCMV, although IFN-I is important in induction and maintenance of a persistent viral infection [18], [19], early IFN-I induction has been shown to decrease viral titers during the first few days of infection [20], [21] and mice lacking the type-I IFN receptor never clear a persistent infection.

LCMV is an enveloped virus containing a bi-segmented, negative strand RNA genome that encodes for four proteins [22]–[24]. The virus nucleoprotein (NP) binds to viral RNA to form the nucleocapsid and associates with the virus polymerase (L protein) to form the virus ribonucleoprotein (RNP) complex that directs virus RNA replication and gene transcription [25], [26]. NP has also been shown to be responsible for the anti-interferon activity of LCMV [27]. The glycoprotein is expressed as a single polypeptide (GPC) that is rapidly cleaved into GP1, GP2 and a stable signal peptide which form a complex at the virus surface that mediates virus receptor recognition and cell entry [28]–[30]. LCMV encodes also a small RING finger protein (Z) that is a bona fide functional matrix protein and driving force of arenavirus budding [31]–[33].

To investigate differences driving events leading to either acute or persistent viral infection, we used infection of mice with Armstrong (Arm) and Cl13 strains of LCMV, which are genetically closely related and share identical CD8+ and CD4+ T cell epitopes but exhibit drastic different phenotypes in their ability to establish persistence. In adult immunocompetent mice Arm causes an acute infection, while Cl13 establishes a persistent one [34]. Genetic and biochemical analysis revealed that only two amino acid differences between these strains of the total 3,356 amino acids encoded by the virus are required for the Cl13-induced persistent infection [35]–[37]. A leucine at position 260 within GP1 allows for a strong binding to the cognate LCMV receptor, α-dystroglycan (αDG) [36], [38]–[40], while a glutamine at position 1079 in the viral polymerase allows for faster and more robust multiplication in vivo in selected cell types such as dendritic cells (DCs) and macrophages [37], [41], [42]. Thus, compared to Arm, Cl13 exhibits a more robust multiplication in plasmacytoid DCs (pDCs) [37], [43], conventional DCs (cDCs) [39], [42], [44], macrophages [41] and fibroblastic reticular cells (FRCs) [45], [46], all cell types essential for establishing an anti-viral immune response required to control and terminate an acute infection.

Early infection of large numbers of pDCs by Cl13 [18], [37], [43], leads to its multiplication in the white pulp [39], [47], disruption of dendritic cell (DC) function [48], [49], disorganization of splenic architecture [50]–[52], upregulation of negative immune regulators [44], [53]–[56] and dysfunctional cytotoxic [57] and helper T cells [58]–[60] necessary for creating and maintaining an immune environment in which Cl13 can persist.

To better understand the basis of how early events dictate the course of whether an infection becomes acute or persistent, we asked 1) how the non-persistent Arm and persistent Cl13 strains of LCMV affect the early induction of the IFN-I response, and 2) how early induction of the IFN-I response affect subsequent events during infection with Arm or Cl13. Our results reported here show that LCMV persistence is dependent on an early dysregulated innate immune response that is associated with rapid viral proliferation. These early events are essential for Cl13 invasion into the splenic white pulp and infection and replication in selected cell types that lead to suppression of the T cell response. On the other hand, an immune response characterized by a subdued cytokine and chemokine signature in the serum and a decreased rate of LCMV multiplication early during infection prevent the establishment of LCMV persistence.

## Results

### Effect of Arm priming on Cl13 persistence

**Figure 1:**
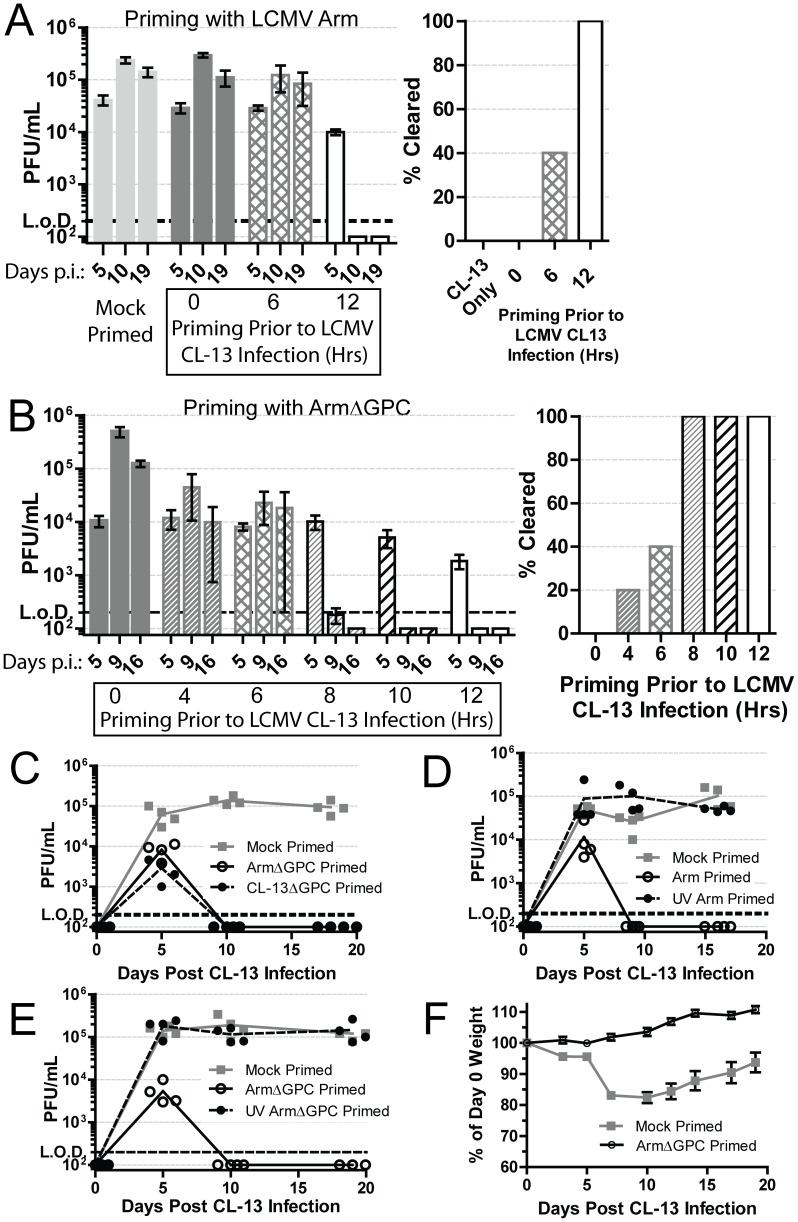
Priming an LCMV infection leads to early clearance. A) Mice (n = 5/group) were either mock (PBS) or Arm (2×10^6^ ffu, i.v.) primed 6 or 12 hours prior to, or concurrently, with Cl13 (2×10^6^ ffu, i.v.) infection. At 5, 10 and 19 days post-infection serum virus titers were determined by plaque assay on Vero cells. The right panel shows mice (%) that had cleared viremia (<200 pfu/ml in serum) by day 19 post-infection Data are representative of three independent experiments. B) Same as A except mice were primed with ArmΔGPC at the indicated intervals. Data are representative of two independent experiments. C) Mice (n = 4/group) were mock (PBS) or ArmΔGPC (2×10^6^ ffu, i.v.) or Cl13ΔGPC (2×10^6^ ffu, i.v.) primed 12 hours prior to Cl13 infection ((2×10^6^ ffu, i.v.) and virus titers in serum determined as in (A) at the indicated times. D+E) Mice (n = 4/group) were mock primed or primed with UV-irradiated or non-irradiated Arm (D) or ArmΔGPC (E). Serum virus titers were determined as in (A) at the indicated time points. F) Mice (n = 19 mice/group) were either mock (PBS) or ArmΔGPC (2×10^6^ ffu/ml) primed 12 hours before infection with Cl13 (1×10^5^ ffu/ml, i.v.) and weighed at the indicated times. Data in C–E are representative of two independent experiments.

Murine infection with immunosuppressive strains of LCMV, such as Cl13, result in higher infection of, and multiplication within, pDCs when compared to mice infected with LCMV strains, such as Arm, that cause acute viral infection terminated by a robust T cell response [18], [36], [37], [39], [43]. To examine whether one of these two phenotypes was dominant over the other, we injected mice with a dose of Cl13 (2×10^6^ i.v.) that causes a persistent infection in adult immunocompetent mice. Concurrently, these mice also received the same dose of Arm. Mice that received both Cl13 and Arm simultaneously did not clear early and had similar serum titers compared to mice receiving PBS and Cl13 (mock primed) (Fig. 1A). These data indicate that the persistent phenotype of Cl13 was dominant over the acute phenotype of Arm. To determine if Arm was able to trigger an immune response capable of blocking the establishment of Cl13 persistence, we primed mice first with Arm before Cl13 infection. Mice primed with Arm (2×10^6^ i.v.) twelve hours prior to Cl13 infection (2×10^6^ i.v.), cleared infection in two weeks despite similar serum titers at day 5 post-Cl13 infection (Fig. 1A). When six hours separated the priming (Arm) dose and Cl13 infection, 40% of mice cleared the infection demonstrating that Cl13 must be able to modify the host immune response early after infection in order to establish a persistent infection in its host.

### Contribution of viral gene expression, replication and propagation to Arm priming-mediated clearance of Cl13

To understand how Arm triggers a primed immune response that facilitated control and clearance of a subsequent infection with Cl13, we used a non-propagating Arm for priming prior to Cl13 infection. This non-propagating recombinant LCMV has the gene for the enhanced green fluorescent protein (EGFP) in place of the gene encoding GPC (ArmΔGPC). Trans-complementation of ArmΔGPC with GPC of Arm results in a single-cycle infectious virus that can infect and replicate in infected cells, but cells infected with this virus cannot produce infectious progeny viruses. Mice primed with ArmΔGPC cleared Cl13 infection within two weeks if the priming dose was administered at least 8 hours prior to Cl13 infection (Fig. 1B). One mouse (of five) cleared the infection when primed four hours before Cl13 infection (Fig. 1B, right panel). Mice primed with ArmΔGPC up to ten hours before Cl13 infection had similar viral titers at day 5 post-infection whether or not they later cleared the infection. When mice were primed at 12 hours before Cl13 infection, viral titers at day 5 were lower than those of mock primed mice (Fig. 1B).

Adult immunocompetent mice infected with Cl13 never clear virus from their kidneys despite undetectable viral titers from all other organs and serum four months post-infection [61]. To assess whether Cl13 had completely cleared from ArmΔGPC primed mice infected with Cl13, we plaqued tissue homogenates from the spleens, livers, lungs, kidneys, hearts, and brains of these animals 18 days post-infection. We did not detect the presence of virus (<200 pfu/gram) in any of these organs. Mice primed with a non-propagating Cl13 virus also cleared the infection suggesting that neither tropism nor differences in Cl13 viral gene expression were responsible for a primed-mediated clearance of Cl13 (Fig. 1C). When the priming agent (either Arm or ArmΔGPC) was UV-inactivated, thereby unable to express viral genes or replicate, Cl13 was able to persist in mice (Fig. 1D – E).

Altogether, these data demonstrate that the priming agent must be able to either express viral genes or replicate, or both, to trigger an immune response capable of clearing a Cl13 infection. Furthermore, viral propagation was not necessary to generate this response as priming with ArmΔGPC induced an immune response that efficiently cleared Cl13.

Infection with a lower dose of Cl13 (1×10^5^ i.v.) does not lead to persistent infection but causes weight loss and a death for less than 25% of infected mice [62]. Priming of mice with a high dose (2×10^6^ i.v.) of ArmΔGPC 12 hours prior to a lower dose (1×10^5^ i.v.) Cl13 infection prevented weight loss and mortality. Mock primed mice (injected with a similar volume of PBS) lost nearly 20% of their original weight (Fig. 1F) and had a 10.5% fatality rate (2/19 mice). These data demonstrate that priming with ArmΔGPC not only leads to clearance of a high dose Cl13 infection, but also prevents the disease associated with a lower dose Cl13 infection.

### Role of CD8+ T cells on Arm priming-mediated clearance of Cl13

**Figure 2:**
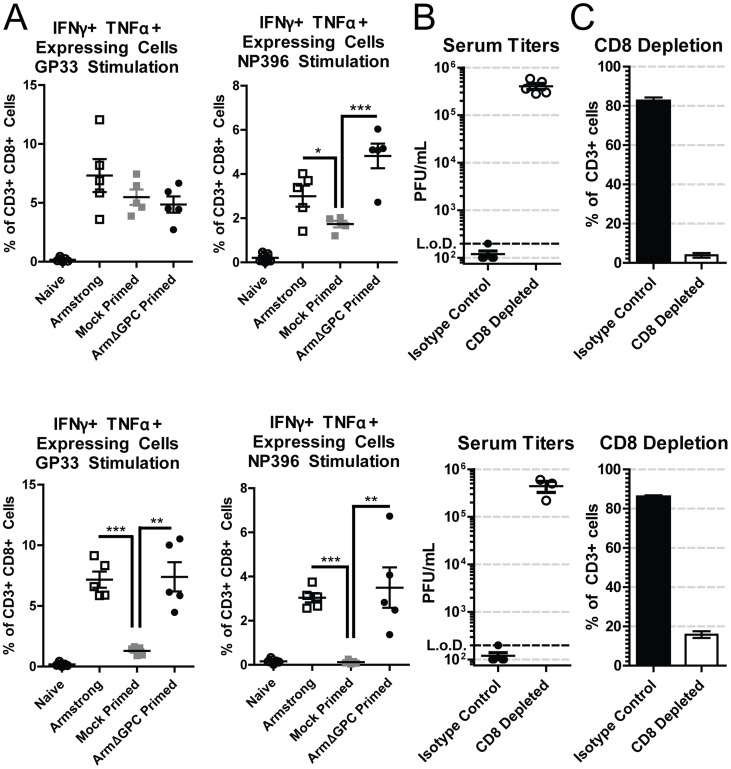
CD8+ T cells are necessary for Arm primed mediated clearance. C57Bl6/J (H-2^b^) mice (n = 5/group) were bled at either day 7 (top panels) or day 11 (bottom panels). A) C57Bl6/J (H-2^b^) mice were infected with Arm (2×10^6^,i.v.), or mock (PBS) or Arm (2×10^6^ ffu, i.v.) primed 12 hours before infection with Cl13 (2×10^6^,i.v.). Blood samples were collected at 7 (top panels) or 11 (bottom panels) days post-infection and after erythrocyte depletion incubated with H-2b restricted peptides GP_33–41_ and NP_396–404_, IL-2 and BrefeldinA. Cells were stained with antibodies against mouse CD3ε, CD8a, IFNγ and TNFα and analyzed by flow cytometry. *p<0.05; **p<0.005; **p<0.0005. B and C) C57Bl6/J mice were primed with ArmΔGPC (2×10^6^ ffu, i.v.) and 12 hours later infected with Cl13 (2×10^6^ ffu, i.v.). Mice received intraperiotneal injections of 0.75 mg/mouse anti-CD8 antibodies at −1 and +1 days following priming. B) Virus titers in serum were determined as in Fig. 1. C) Percentages of CD8+T cells from total CD3+ cells in the blood were determined by staining erythrocyte depleted blood with antibodies against mouse CD3 and CD8 and analyzed by flow cytometry. Two mice in the CD8 depleted group died at day 10.

T cells from adult immunocompetent mice infected with Cl13 have a decreased function (T cell exhaustion) that leads to their inability to clear the infection [57]. Viral titers in serum at five days post-infection were similar in mock and ArmΔGPC primed Cl13 infected mice infected with Cl13 (Fig. 1B, mice primed 8 and 10 hours prior to Cl13 infection). We therefore tested whether a cytotoxic T cell (CTL) response was responsible for clearance in primed mice. CTL function was assayed by incubating splenocytes isolated from mock or ArmΔGPC primed C57Bl6/J (H-2^b^) mice with immunodominant H-2D^b^-restricted LCMV-derived peptide epitopes GP_33–41_ and NP_396–404_, which are conserved between Arm and Cl13, and assessing intracellular expression of interferon-γ (IFNγ) and tumor necrosis factor-α (TNFα) in CD8+ T cells by flow cytometry. We found that CTLs from Cl13 infected mice primed with ArmΔGPC had a robust T cell response to GP_33–41_ and NP_396–404_ LCMV-derived peptides comparable to CTLs from mice infected with Arm alone (Fig. 2A). In comparison, significantly fewer CTLs isolated from mock primed Cl13 infected mice expressed IFNγ and TNFα, a phenotye typical of a persistent infection. Only T cells isolated seven days post Cl13 infection and stimulated with GP33 did not show significant differences, however significant differences were seen between these groups at 11 days post Cl13 infection.

To further ensure that CD8+ T cells were responsible for viral clearance in this model we conducted depletion experiments. Mice were depleted of CTLs using CD8 specific antibodies, primed with ArmΔGPC and infected with Cl13 12 hours after priming (Fig. 2B and 2C). Mice treated with non-relevant isotype control antibodies cleared a primed Cl13 infection while primed Cl13 infected mice depleted of CTLs were viremic at days 7 and 11 (Fig. 2B) confirming that CTLs were responsible for clearing LCMV. CD8+ T cell depletion was very efficient at both days 7 and 11 post Cl13 infection (Fig. 2C).

### Cytokine and chemokine expression during establishment of Cl13 persistence

Clearance of Cl13 from ArmΔGPC primed mice was mediated by CTLs, raising the possibility that the introduction of viral antigens (via ArmΔGPC administration) 8 hours prior to Cl13 infection led to an 8 hour advantage in T cell generation, and provided enough time to tip the balance in favor of viral clearance. Under this scenario, we would expect a similar, but earlier induction of the innate immune response in primed mice compared to mock primed mice in the first 24 hours after Cl13 infection. To examine this possibility we measured expression levels of a panel of cytokines in serum samples collected at several times after priming and infection.

**Figure 3:**
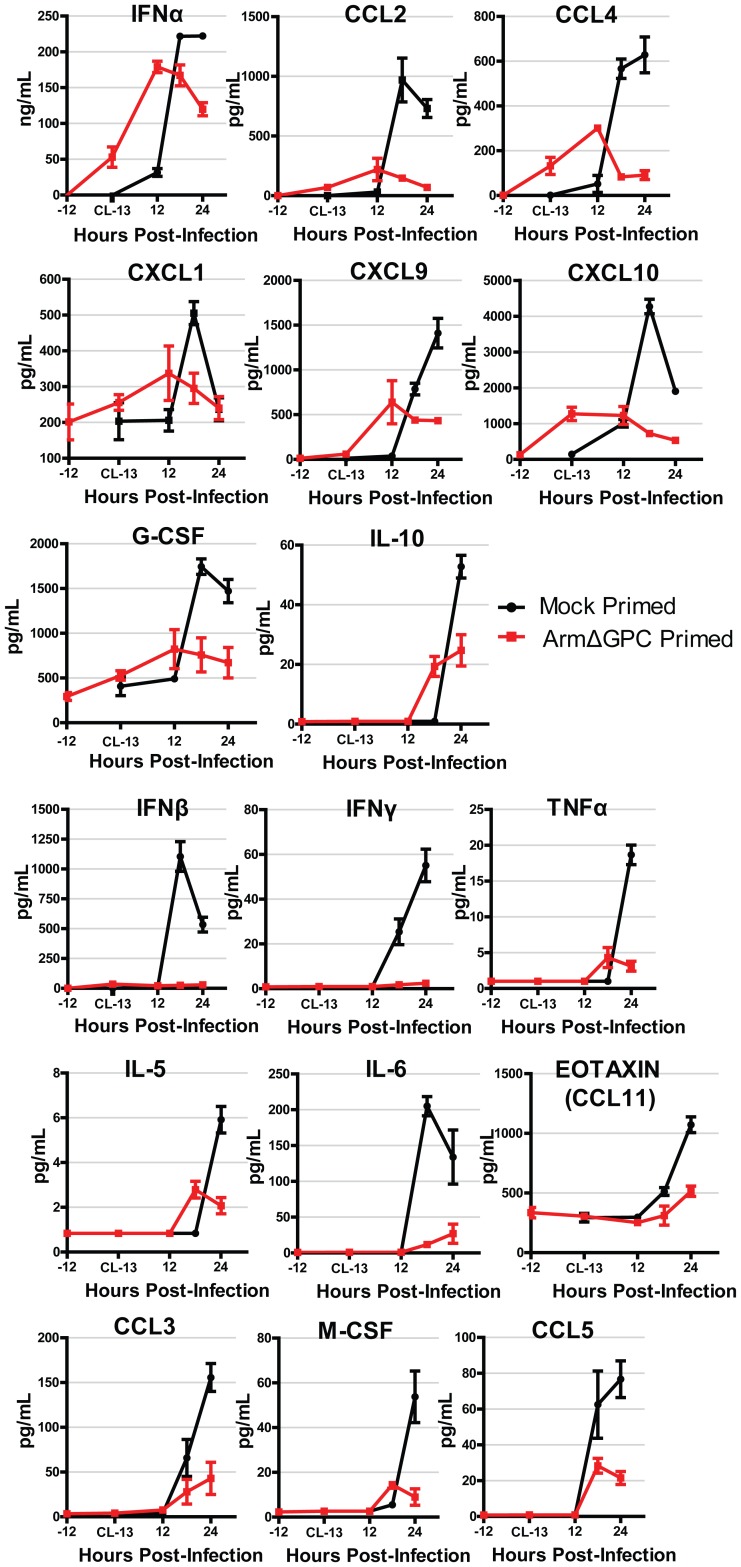
Cytokine responses during primed and mock primed Cl13 infected mice. C57Bl6/J mice (n = 4/group) were mock (PBS) or ArmΔGPC (2×10^6^,i.v.) primed and 12 hours later infected with Cl13 (2×10^6^,i.v.). Standard ELISAs (IFNα and IFNβ) and multiplex ELISAs were performed on serum samples taken at the indicated time points. Red and black lines correspond to values from ArmΔGPC or mock, respectively, primed mice. Data for cytokines that were not detected by the multiplex ELISA and those for which there was no discernible difference were not graphed.

We found that cytokine levels in the serum of Cl13-infected mice varied substantially between primed and mock primed mice. These differences fall roughly into two groups. The first group (Fig. 3, top three rows) was defined by cytokines that were induced in both mock and ArmΔGPC primed mice. Within this group, IFNα, CCL4, CXCL1, and CXCL10 were upregulated following ArmΔGPC priming but prior to infection with Cl13, suggesting that these cytokines were upregulated not only upon Clone 13 infection but also by the priming agent. Overall, for each cytokine, expression was higher at its peak in mock primed compared with ArmΔGPC primed Cl13 infected mice.

The second group (Fig. 3, bottom 3 rows) was defined by cytokines that were expressed at significantly lower levels in ArmΔGPC primed compared to mock primed Cl13 infected mice suggesting that priming with ArmΔGPC affected the ability of these cytokines to be upregulated by Cl13 infection. Interestingly, IFNα and IFNβ expression fall into these separate groups. Whereas ArmΔGPC primed and mock primed Cl13 infected mice expressed significant amounts of IFNα which peaked 6–12 hours earlier than in mock primed Cl13 infected mice, only negligible amounts of IFNβ were detected in the serum of primed mice during the first 24 hours following Cl13 infection. This difference between IFNα and IFNβ is similar to what has been observed between Arm and Cl13 infected mice [18]. Importantly, results shown in Fig. 3 indicate that an immune response in mice that clear Cl13 infection within two weeks is characterized by lower overall expression levels of cytokines and chemokines compared to mock primed mice. Conversely, a dysregulated innate immune response early after Cl13 infection is associated with the establishment of viral persistence. In addition, while we have previously observed that in Cl13-infected mice IFN-I signaling was responsible for the upregulation of a wide variety of cytokines and chemokines [18], in ArmΔGPC primed mice, early IFNα expression is associated with an overall decrease in cytokine and chemokine expression compared to mock primed Cl13 infected mice.

### Role of the immune environment in viral antigen expression and distribution in the spleen

**Figure 4:**
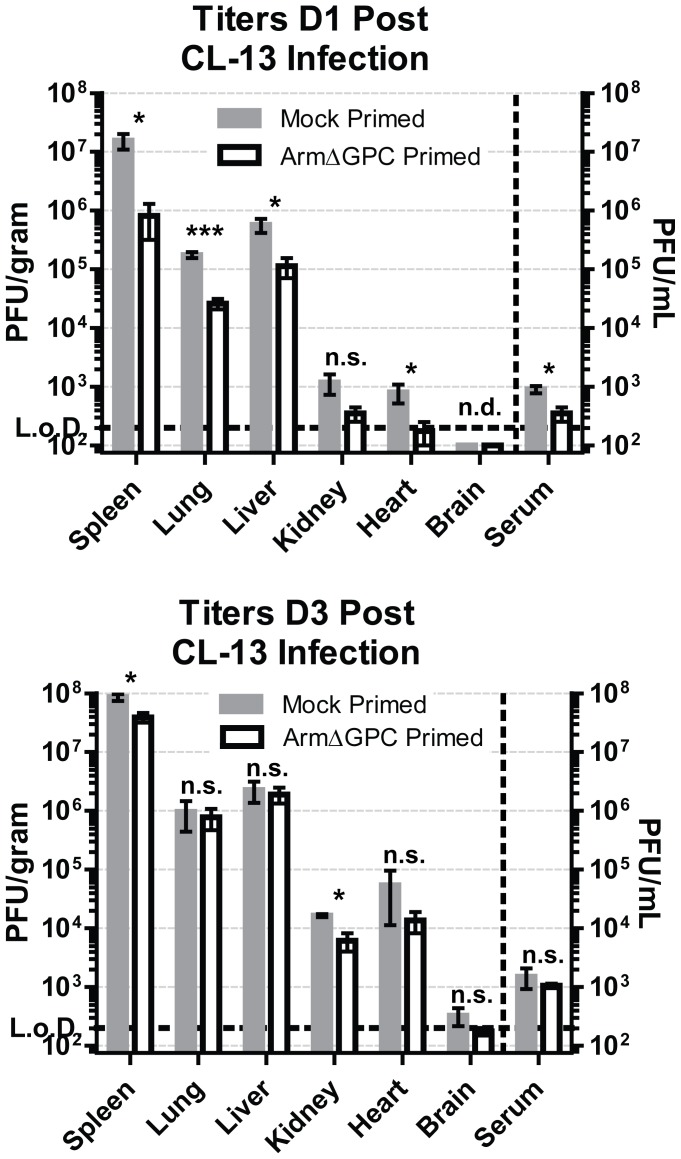
Kinetics of viral propagation at days 1 and 3 after Cl13 infection. C57Bl6/J mice (n = 4/group) were mock (PBS) primed or ArmΔGPC (2×10^6^,i.v.) primed and 10 hours later infected with Cl13 (2×10^6^,i.v.)). Organs were harvested at 24 (top panel) and 72 (bottom panel) hours post-infection and homogenates prepared in RPMI containing 10% FBS. Virus titers in clarified homogenates were determined as in Fig. 1. Statistical analysis was done using t-tests of log transformed values. NS, not significant; ND, none detected; *p<0.05; **p<0.005; **p<0.0005. Graphs are representative of three independent experiments.

We found that ArmΔGPC priming 8–10 hours before Cl13 infection did not affect virus levels in serum of Cl13-infected mice at day 5 post-infection (Fig. 1A and B), but early induction of IFNα in primed mice may have affected viral multiplication at earlier infection times. To examine this, we titered virus from the organs and serum at 24 and 72 hours after Cl13 infection of mock primed mice and mice primed with ArmΔGPC 10 hours prior to Cl13 infection. After 24 hours of infection, we observed a significant decrease (∼1 log_10_) in LCMV titers in most organs tested (Fig. 4, top panel). However, by three days post-infection, titers between both groups were similar (Fig. 4, bottom panel).

**Figure 5:**
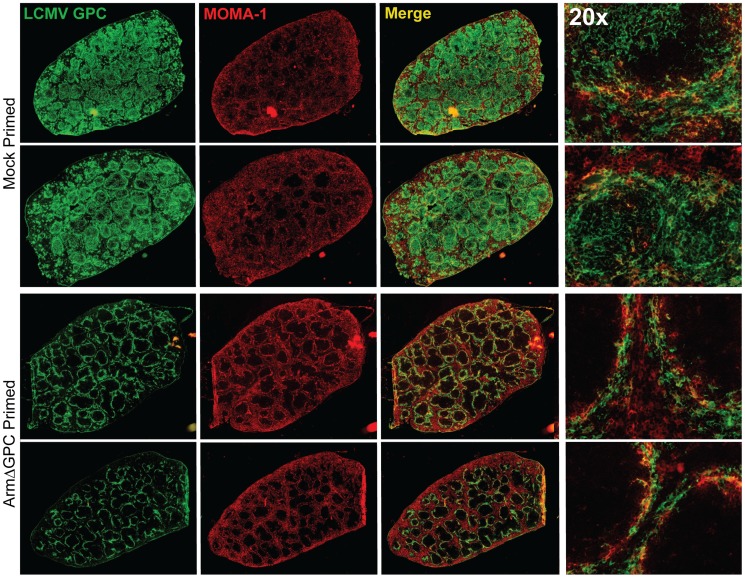
Differential localization of viral antigen in spleens of primed and mock primed Cl13 infected mice. C57Bl6/J mice (n = 3/group) were primed, infected and spleens were harvested as in Fig. 4 at 72 hours post-infection. Spleens were flash frozen in OCT and 6–8 µm sections were prepared and stained with a guinea pig serum to LCMV GPC (green). Splenic microarchitecture was assessed by visualization of mellalophilic macrophages using anti mouse MOMA-1 antibodies (red). Sections from two mice/group are shown here and are representative of the group as a whole. Data are from one of two independent experiments.

At three days post-infection, Cl13 and Arm are largely found within the white and red pulp, respectively, in the spleen [39], [47] and dissemination of Cl13 in to the white pulp of the spleen has been associated with the ability of Cl13 GPC to interact with the host receptor αDG [38]. Viral titers in total spleen homogenates were similar at three days post-infection in ArmΔGPC and mock primed mice infected with Cl13 (Fig. 4). To examine possible differences in Cl13 distribution between ArmΔGPC and mock primed mice, we prepared spleen tissue sections from mice that were mock primed and ArmΔGPC primed 10 hours before Cl13 infection at three days post-infection and stained them with fluorescently conjugated antibodies against the viral GPC and MOMA-1, a marker of metallophilic macrophages found on the inner border of marginal zones adjacent to the white pulp. Viral antigen in spleens from ArmΔGPC primed Cl13 infected mice was found largely bordering the white pulp in marginal zone regions while viral antigen in spleens of mock primed Cl13 infected mice was found in both marginal zones and in the white pulp of the spleen (Fig. 5). These data show that while ArmΔGPC primed and mock primed Cl13 infected mice had similar viral titers, there were clear differences in the localization of LCMV GPC. Together with data from Fig. 3, our results strongly suggest that a dysregulated immune response early during Cl13 infection is likely an essential component to the movement of Cl13 out of the marginal zones and into the splenic white pulp.

**Figure 6:**
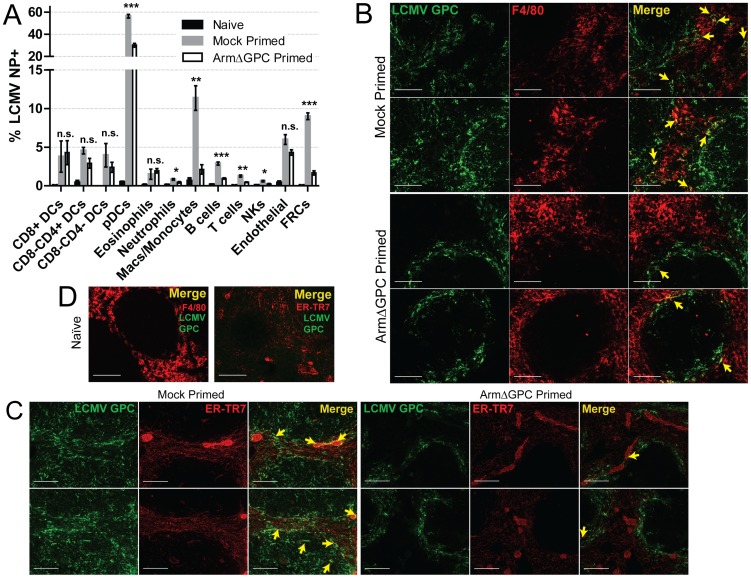
Priming with ArmΔGPC changes cellular tropism during Cl13 infection. C57Bl6/J mice (n = 4/group) were primed and infected as in Fig. 5 and spleens were harvested at 72 hours post-infection. A) Spleens were incubated with 1 mg/mL collagenase D/100 µg DNaseI and manually dissociated through 100 µm nylon screens. Cells were identified by flow cytometry through staining and gating with the following fluorescently conjugated anti-mouse antibodies: DCS: lin-(CD90.2/CD19), CD11c^high^; pDCs: lin-, CD11c+, SiglecH+, PDCA-1+, B220+; Eosinophils: lin-, CD11b+, Ly6G-, Ly6c^low^, SSC-A^high^; Neutrophils: lin-, CD11b+, F4/80-, Ly6G+; Macs/Monocytes: lin-, CD11b+, Ly6G-, Ly6c+, F4/80+; B cells: B220+, CD3-, CD8-, NK1.1-; T cells: B220-, NK1.1-, CD3+, CD8+ or CD4+; NK cells: CD3^low^, B220-, NK1.1+; Endothelial cells: lin-, CD45-, gp38+, CD31+; FRCs: lin-, Cd45-, gp38+, CD31-, CD35^low^. Representative data from one of two independent experiments are shown with SEM calculated and displayed using GraphPad Prism software. Statistical analysis (unpaired t-test) is shown between mock primed and ArmΔGPC primed Cl13 infected groups. NS, not significant; *p<0.05; **p<0.005; **p<0.0005. B–D) Spleens treated as in Fig. 5 and 6 µm sections were stained with guinea pig serum to LCMV GPC (Green) and antibodies used to visualize mature macrophages (F4/80, red, B and D) and fibroblastic reticular cells (ER-TR7, red, C and D). Yellow arrows indicate areas of costaining. The two examples from each staining are from different mice. D) Spleens of naïve mice are shown for comparison and specificity of LCMV serum. White bars indicate 100 µm. All micrographs are representative examples of micrographs from two independent experiments.

To assess whether cellular tropism differed between mice primed with ArmΔGPC 10 hours before Cl13 and mock primed Cl13 infected mice, we made single cell suspension preparations from spleens of these mice three days after Cl13 infection and quantified the presence of viral NP in various cell types by flow cytometry. Highly significant differences were seen in plasmacytoid DCs (pDCs) (mock primed: 56.4±1.6; ArmΔGPC primed: 30.2±1.7, p = 0.00003), macrophages/monocytes (mock primed: 11.4±1.6; ArmΔGPC primed: 2.1±0.6, p = 0.0017) and FRCs (mock primed: 9.0±0.4; ArmΔGPC primed: 1.7±0.3, p = 0.000007) from primed and mock primed Cl13 infected mice (Fig. 6A). These cell types have been associated with persistent viral infection and essential for a proper anti-viral immune response [41], [43], [45], [46], [63], [64]. Additionally, we observed significant differences in T (mock primed: 1.3±0.2; ArmΔGPC primed: 0.5±0.04, p = 0.001) and B cells (mock primed: 2.9±0.2; ArmΔGPC primed: 0.9±0.08, p = 0.0001). Higher numbers and percentages (though low overall) of infected T and B cells may be due to the increased presence of virus in splenic white pulp (Fig. 5) where a vast majority of T and B cells reside.

We observed increased costaining of LCMV NP with F4/80, a marker of mature macrophages, in mock primed Cl13 infected mice (Fig. 6B) three days post-infection. This is coincident with invasion of LCMV into the red pulp, where as GPC staining was restricted mostly to the marginal zones in spleens of ArmΔGPC primed Cl13 infected mice. Additionally, we observed increased costaining of LCMV GPC with ER-TR7, a marker of stromal cells in secondary lymphoid tissue, particularly FRCs. Notably, some (but not all) of the reticular fibroblasts lining the red pulp sinus contained viral antigen as well as some FRCs present in the white pulp in the spleens of mock primed Cl13 infected mice three days post-infection (Fig. 6C). Fig. 6D shows the architecture and antibody staining of spleens from naïve (uninfected) mice for comparison. Together, our data show that viral propagation into red and white pulp was coincident with a wider viral cellular tropism. Viral antigen in the spleens of ArmΔGPC primed Cl13 infected mice was primarily limited to areas within and near metallophillic macrophages that typically line the inner portion of the marginal sinus.

### Role of cytoplasmic RNA sensing on Arm priming-mediated clearance of Cl13

**Figure 7:**
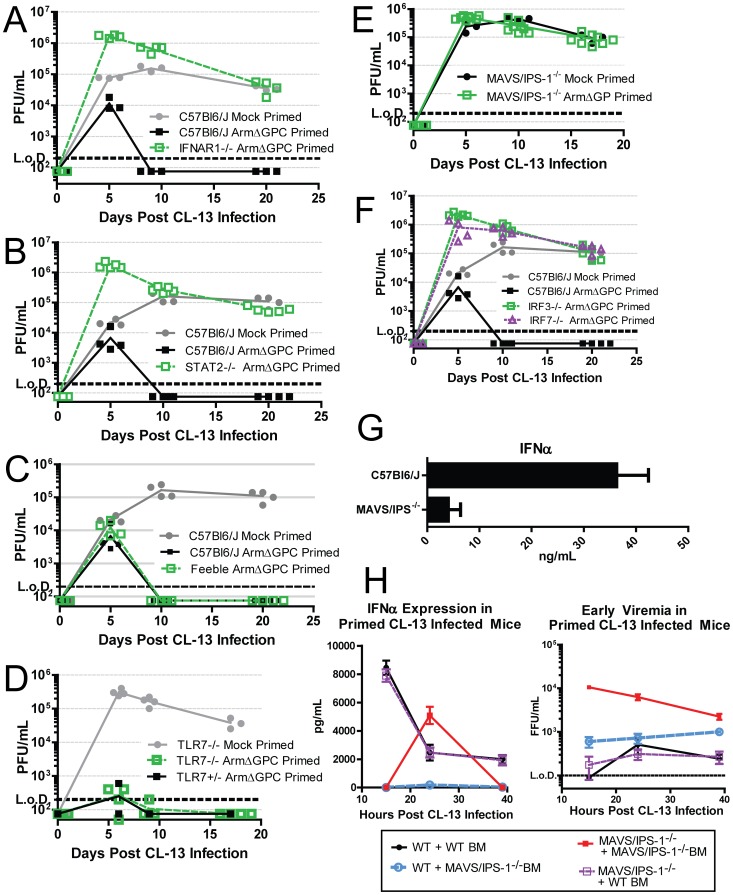
The role of IFN-I during a primed Cl13 infection. A–F) Mice were mock (PBS) primed or ArmΔGPC (2×10^6^,i.v.) primed 12 hours prior to infection with Cl13 (2×10^6^,i.v.). At the indicated times virus titers in serum were determined as in Fig. 1. G) Mice were infected with Cl13 (2×10*^6^* ffu, i.v.) and at 24 hours post-infection serum levels of IFNα were measured by ELISA. H) Ly5.1 C57Bl6/J mice and MAVS/IPS-1^-/-^ mice were γ-irradiated and 5×10^6^ bone marrow cells from either Ly5.1 and MAVS/IPS-1^-/-^ were injected i.v. into γ-irradiated mice. Mice were fed antibiotic feed for 5 weeks and 8 weeks post γ-irradiation, mice were primed with ArmΔGPC and infected with Cl13 12 hours later. Serum was taken at the indicated time points and assayed for IFNα by ELISA (left) or plaqued on Vero cells (right) (n = 7/group). All data shown are representative of at least 2 independent experiments.

Mice lacking the IFN-I receptor (IFNAR1^-/-^) failed to clear a primed Cl13 infection (Fig. 7A) confirming that the early IFN-I signaling is required for early clearance. Downstream of IFNAR1 engagement with IFN-I, signal transducer and activation of transcription 2 (STAT2) forms a complex with STAT1 and IRF9 to form the transcription factor, ISGF3. STAT2 null mice also failed to clear a primed Cl13 infection (Fig. 7B).

Because pDCs are prodigious producers of type-I IFN, we tested whether mice that express a hypomorphic Slc15a4 (termed “feeble”), a protein essential to IFN-I production in pDCs upon stimulation of TLRs, can clear an ArmΔGPC primed Cl13 infection. The phenotype in feeble mice, is restricted to pDCs and has no effect on conventional DCs [20], [63]. Feeble mice cleared the primed infection similarly to its wild-type counterparts (Fig. 7C) indicating that release of IFN-I from pDCs due to TLR stimulation is not necessary to clear a primed infection.

IFN-I is induced upon stimulation of PRR by PAMPs. In the case of LCMV, viral RNA is likely a primary PAMP due to the inability of mice that lack TLR7, which recognizes ssRNA [65], [66], to clear Cl13 infection [67]. However Cl13 infected TLR7^-/-^ mice primed with ArmΔGPC have undetectable viral titers after two weeks of infection (Fig. 7D) suggesting that the IFN responsible for clearance was not generated though TLR7 stimulation. Viral RNA can also be sensed by the cytosolic PRRs RIG-I and MDA-5, both of which signal through the adaptor MAVS/IPS-1 leading to nuclear translocation of IRF3, upregulation of IRF7 expression and subsequent induction of IFN-I gene expression [9]. MAV/IPS-1^-/-^ mice failed to clear ArmΔGPC primed Cl13 infected mice early (Fig. 7E). Moreover, IRF3 and IRF7 expression was also required for priming mediated clearance (Fig. 7F). These data indicate that IFN induced through cytoplasmic RNA sensing of ArmΔGPC was responsible for clearance of primed Cl13 infected mice.

### Role of MAVS/IPS-1 signaling in hematopoietic and non-hematopoietic cells in production of IFNα restricted early viral replication in primed mice

Serum IFNα was measured in Cl13 infected wild-type (WT) and MAVS/IPS-1^-/-^ mice. Most of the IFNα present in serum 24 hours post-infection was due to signaling through cytoplasmic RNA sensors (Fig. 7G). Cytoplasmic RNA sensors are found in both hematopoetic and non-hematopoetic cells. To determine the origin of the serum IFNα, we generated bone marrow chimeric mice between MAVS/IPS-1^-/-^ and Ly5.1 congenic WT mice. Chimeric mice displayed >95% reconstitution as measured by analyzing the presence of CD45+Ly5.1+ and CD45+Ly5.1- cells in blood by flow cytometry. Eight weeks after bone marrow transfer, mice were primed with ArmΔGPC twelve hours prior to Cl13 infection. Serum samples were taken at 15, 24 and 39 hours post-infection and analyzed by ELISA for IFNα. MAVS/IPS-1^-/-^ mice reconstituted with WT bone marrow showed similar levels of serum IFNα as WT mice reconstituted with WT bone marrow (Fig. H, left panel), revealing that MAVS/IPS-1 signaling in hematopoietic cells is responsible for circulating IFNα.

Interestingly, we observed a spike of IFNα in the serum of MAVS/IPS-1^-/-^ mice that was not present in other groups. Consistent with previous findings [18], in the absence of IFN-I, viral titers were significantly higher than those measured in WT controls (Fig. 7A, 7B and 7F). We posited that increased viral titers in these mice would result in higher levels of virally derived TLR agonists that would trigger a more robust TLR response leading to the spike in IFNα seen in this group but not in others. Indeed, when we measured the serum titers of these chimeric mice, viremia was ten-fold higher in MAVS/IPS-1^-/-^ mice reconstituted with MAVS/IPS-1^-/-^ bone marrow at 15 and 24 hours post Cl13 infection compared to any other group. Decreased viremia in these mice at 39 hours post-infection followed the spike of IFNα observed in the serum at 24 hours.

Although no IFNα was detected in the serum of WT mice reconstituted with MAVS/IPS-1^-/-^ bone marrow, viral titers were lower in these mice compared to MAVS/IPS-1^-/-^ mice reconstituted with MAVS/IPS-1^-/-^ bone marrow suggesting that non-hematopoietic cells contributed to early viral control even if they did not contribute to measurable IFNα in the serum. Altogether, these data indicate that the majority of IFNα produced early in primed mice responsible for early clearance of Cl13 is produced by hematopoetic cells through triggering of cytoplasmic RNA sensors.

## Discussion

In this paper we report how early events affect the establishment of a persistant infection by comparing the outcome of infection of mice with the Cl13 strain of LCMV under two different immune environments. For this we used a model of LCMV infection where mice were first primed with a non-propagating LCMV (ArmΔGPC), or mock-primed with PBS, to examine how a subsequent infection with the immunosuppressive strain Cl13 was able to overcome the effects of virally induced cytoplasmic RNA sensing in primed mice.

Despite encoding a NP capable of counteracting IFN-I induction, the LCMV Arm isolate triggers a potent IFN-I response followed by an effective T cell response that results in viral clearance in lieu of a persistent infection. In contrast, the LCMV Cl13 isolate overcomes this response through rapid and robust multiplication that leads to immune dysregulation through the upregulation of cytokines and chemokines, dissemination from marginal zones, infection of important immune cells, and hyporesponsive T cell response. Such “exhausted” T cells are unable to clear the infection, thereby creating an environment favorable to viral persistence. Notably, some mice primed with the non-persistent Arm strain of LCMV as early as 4–6 hours prior infection with Cl13 were able to control and clear Cl13 while all mice primed after 8 hours prior to infection were able to clear a Cl13 infection.

Hence, a narrow temporal window exists between the host's ability to mount an appropriate anti-viral immune response through triggering of cytoplasmic PPR and a dysregulated immune response caused by rapid viral propagation. When inoculated concurrently, Cl13 overcame the innate immune response triggered by cytoplasmic PRRs induced by the priming agent, a response also presumably generated by Cl13 itself. However, if this antiviral innate immune response was triggered as early as 4 hours prior to infection, then Cl13 could not overcome the response in some mice while all mice primed 8 hours prior to infection cleared the infection within two weeks. Under these conditions, viral growth slowed, as evidenced by lower viral titers in serum and organs after 24 hours of infection. Despite the initial comparative decrease in viral titers between primed and unprimed mice, viral titers in primed mice recovered to levels of unprimed mice after 3 days of infection but persistent infection did not occur. This suggests that antigen load is not solely responsible for T cell exhaustion. However, the possibility remains that either a high antigen load earlier than day 3 post-infection or high antigen load in distinct areas of the spleen affects T cell responses.

Infection of white pulp area of the spleen, a region where important interactions between cells of the innate and adaptive immune response occur, is a hallmark of persistent LCMV infection [39], [47], and eventually leads to the disruption of splenic architecture [50]–[52]. LCMV initially infects the marginal zone and it was unclear what determined movement of LCMV from marginal zones to the white pulp. LCMV isolates that cause persistent infection uniformly bind αDG with 2 to 3 orders higher affinity than isolates that cause acute infection [30], [39], [68], which led to the conclusion that cellular tropism is a main factor mediating the movement of LCMV into the white pulp. However, our results have uncovered a critical contribution of the immune environment in restricting LCMV to marginal zone areas. Therefore, the combination of both cellular tropism through GP-αDG interactions and the immune environment via the increased expression of chemokines, likely facilitates viral invasion into the white pulp and infection of relevant cell types.

LCMV NP is a robust inhibitor of cytoplasmic RNA sensing pathways [15]–[17], [27], [69], but published data showed a robust IFN-I induction upon LCMV infection [18], [21], [69]. Here we present evidence that cytoplasmic pathogen sensing mediated through the signaling molecule MAVS/IPS-1 plays a critical role in production of IFN-I following infection with LCMV of adult immunocompetent mice. This finding suggests that NP is only partially effective in preventing MAVS/IPS-1-mediated induction of IFN-I by infected cells during LCMV infection. It is also possible that in vivo DCs might sense LCMV-infected cells by a mechanism that is independent of LCMV multiplication in DCs, resulting in production of IFN-I by DCs that are not productively infected [70], [71]. The anti-IFN-I activity of NP may serve to dampen the IFN-I response to levels that can be overcome by strains of LCMV, like Cl13, capable of multiplying very rapidly in vivo.

The IFN-I response to LCMV infection also has a proviral effect as signaling through IFNAR is necessary for upregulation of negative immune regulators, disruption of splenic architecture and maintaining viral persistence [18]. LCMV Cl13 likely overcomes the initiation of an effective anti-viral immune response not by abrogating the IFN-I response, but by overstimulating the immune response through rapid growth in and near hemotopoetic cells, which are responsible for initiating an appropriate anti-viral immune response through IFN-I signaling. Counterintuitively, we observed that an early induction of IFN-I mediated by ArmΔGPC priming led to the lack of the upregulation of cytokines that are dependent on IFN-I signaling. The rapid increase in viral titers in mock primed Cl13 infected mice likely contributed to immune simulation and dysregulation through continuous triggering of innate sensors. Indeed, we observed an increase in IFN in the absence of cytoplasmic RNA sensing that was coincident with high viremia whereas we did not observe this later induction of IFN-I in infected mice with lower LCMV serum titers.

In conclusion, our data indicate that interactions between virus and host within hours of infection dictate the course of a persistent infection. The ability of LCMV to persist depends on counteracting the effects of a programmed early immune response mediated by cytoplasmic RNA sensors likely through rapid viral replication. The resultant dysregulated immune response is critical for LCMV invasion into the white pulp of the spleen and infection of pDCs, macrophages, and FRCs, cells necessary for an effective anti-viral immune response. The better we understand how these early immunological events affect the establishment of a persistent viral infection, the more likely the development of more efficacious vaccines for persistent viral infection and development of anti-viral therapies at various stop points in this cycle.

## Materials and Methods

### Ethics statement

Husbandry and handling of mice conformed to guidelines set forth by the National Institutes of Health Guide for the Care and Use of Laboratory Animals and the Department of Animal Resources at TSRI. Experiments involving mice described in this manuscript were performed in an AAALAC accredited vivarium at The Scripps Research Institute (TSRI) (Vertebrate Animal Assurance No. A3194-01) and were approved by TSRI's Animal Care and Use Committee (Protocol #09-0098).

### Mice, virus and in vivo infection

C57Bl6/J mice were purchased from the Rodent Breeding Colony at the Scripps Research Institute (TSRI). MAVS/IPS-1^-/-^ mice were generously provided by Michael Gale (University of Washington). IRF3^-/-^ and IRF7^-/-^ mice were generously provided by Tadatsugu Taniguchi (University of Tokyo). Mice were infected intravenously with 2×10^6^ focus-forming units (FFUs) of virus unless otherwise indicated. Priming of mice with either Arm or ArmΔGPC at 2×10^6^ FFUs occurred 10–12 hours before Cl13 infection unless otherwise indicated. Mock primed mice received an injection of PBS of equal volume and at the same time as the Arm or ArmΔGPC primed mice. Quantification of viremia was conducted by drawing blood from the retro-orbital sinus of mice under isofluorine anesthesia and isolating serum at 6000 rpm for 10 minutes on a tabletop microcentrifuge. Organs were harvested from euthanized mice and homogenized in RPMI containing 10% FBS at a volume that corresponded to the weight of each organ. Viral titers were obtained by plaque assay on Vero cells with either serum or clarified homogenates.

Virus stocks were generated through passage on BHK-21 cells. Non-propagating viruses were generated using reverse genetic technology [72] and passaged on BHK-21 cells transfected with plasmids encoding for LCMV GPC. Titers of viral stocks were acquired by fluorescence focus assay as described [73]. UV-inactivation was performed by direct exposure to UV by incubation on a transilluminator (Ultra-Violet Products, Inc.) for 30 minutes. Only UV inactivated viral stocks that displayed undetectable viral titers (<100 ffu/mL) were used.

### Cell lines, antibodies, and reagents

Vero cells (African green monkey kidney cells originally acquired from the American Type Culture Collection ∼40 years ago) were propagated in Eagles MEM (Gibco) supplemented with 7% FBS, 100 g/mL penicillin/streptomycin (Gibco), 2 mM L-glutamine (Gibco). BHK-21 cells were grown 10% FBS, 100 g/mL penicillin/streptomycin, 2 mM L-glutamine, 7% tryptose phosphate broth solution (Sigma), and 0.56% glucose (wt/vol). The following anti-mouse antibodies were purchased from BD Biosciences: PE CD4 (RM4.5), PE IFNγ, PerCP-Cy5.5 CD3 (145-2c11), PE Ly6G (1A8), and PerCP-Cy5.5 NK1.1 (145-2c11). The following anti-mouse antibodies were purchased from eBioscience: FITC TNFα (MP6-X722), PE SiglecH (440C), PerCP-Cy5.5 CD11c (N418), PerCP-Cy5.5 Ly6c (HK1.4), e450 and APC CD8a (53–6.7), e450 B220 (RA3-6B2), e450 and PE-Cy7 CD90.2 (53–2.1), e450 and PE-Cy7 CD19 (1D3), PE-Cy7 CD3 (145-2c11), PE-Cy7 CD21/CD35, PE-Cy7 CD11b (M1/70), APC and APC-Cy7 CD45 (30F11), APC CD31 (390), and APC PDCA-1 (927). The following anti-mouse antibodies were purchased from BioLegend: PE gp38 (8.1.1), PE-Cy7 CD8a (53–6.7), and APC F4/80 (BM8). Anti-LCMV NP antibody (VL-4) was purchased from BioXcell and conjugated to Alexa Fluor 488 using antibody labeling kit from Life Technologies.

### Peptide stimulation and flow cytometry

As described previously [54], blood was harvested and erythrocyte depleted. Cells were then incubated with H-2b restricted epitopes LCMV GP_33–41_ and NP_396–404_ along with 50 U/mL IL-2. After 1 hour, 1 mg/mL Brefeldin A was added. After an additional 5 hours, cells were harvested, incubated with antibodies against CD16/32 (Fc block), stained with antibodies against CD3ε and CD8α, fixed, permeablized (BD Cytofix/Cytoperm Kit) and subsequently stained with fluorescently conjugated antibodies against IFNγ and TNFα.

For quantification of splenocytes harboring viral nucleoprotein, spleens were harvested, incubated with 1 mg/mL collagenase D/100 µg DNaseI and dissociated as above. Cells were incubated with antibodies against CD16/32 (Fc block), stained with the indicated antibodies to identify various cell types, fixed, permeablized, and incubated with fluorescently conjugated antibodies against LCMV NP (VL-4). Flow cyotometry was performed using a LSR II (Becton-Dickinson) and subsequent analyzed with FlowJo software (TreeStar, Inc.) P values are from t tests calculated using Microsoft Excel.

### Quantification of cytokines and chemokines

IFNα and IFNβ quantification from serum was performed using VeriKineTM Mouse IFNα and IFNβ ELISA Kits (R&D Systems). Levels of all other cytokines and chemokines were analyzed using 32-Plex multiplex ELISA (Millipore).

### Fluorescence microscopy

Spleens were harvested, flash frozen in OCT (Tissue-Tek), and cut into 6–8 µm sections. Sections were fixed in 4% paraformaldehyde, blocked with 10% FBS and stained overnight at 4°C with rat anti-mouse MOMA-1 (ab51814) at 1∶200, rat anti-mouse ER-TR7 (ab51824) at 1∶200, or rat anti-mouse F4/80 (BM8) at 1∶200 and guinea pig anti LCMV GP serum (1∶1000). Sections were washed and incubated with 1∶200 dilutions of AlexaFluor 488-conjugated anti-guinea pig IgG antibodies (Invitrogen) and Alexa Fluor 568 anti-rat IgG (Invitrogen) followed by subsequent washes and mounting with medium from Vector Laboratories. Sections were visualized with a Zeiss Axiovert S100 immunofluorescence fitted with an automated xy stage and an Axiocam color digital camera. Mosaic micrographs were taken with a 5x objective and assembled using Axiovision Software (Zeiss). All other images were taken with a 20x objective.

### Bone marrow chimeras

Ly5.1 congenic C57Bl6/J mice and MAVS/IPS-1^-/-^ mice were γ-irradiatd (1000 rads) and kept on antibiotic feed for 5 weeks. Eight weeks after irradiation, mice were primed with 2×10^6^ ffu ArmΔGPC and infected with 2×10^6^ ffu of Cl13 12 hours later.

### Data analysis

All graphs were made using GraphPad Prism software with SEM calculated and displayed. Significance was calculated using either GraphPad Prism software or Microsoft Excel as indicated.
